# rTMS-induced neuroimaging changes measured with structural and functional MRI in autism

**DOI:** 10.3389/fnins.2025.1582354

**Published:** 2025-05-08

**Authors:** Xiaodong Kang, Kai Chen, Fei Wang, Linyi Mu, Zengzhen Lei, Rufei Zhang, Zedong Wang, Tao Zhang

**Affiliations:** ^1^Affiliated Rehabilitation Hospital of Chengdu University of Traditional Chinese Medicine/Sichuan Provincial BAYI Rehabilitation Center, Chengdu, China; ^2^Mental Health Education Center and School of Big Health Management, Xihua University, Chengdu, China; ^3^School of Computer and Software, Chengdu Jincheng College, Chengdu, China; ^4^The People's Hospital of Baiyun District Guangzhou, Guangzhou, China

**Keywords:** repetitive transcranial magnetic stimulation, autism spectrum disorders, voxel-based morphometry, functional connectivity, neuroimaging

## Abstract

**Introduction:**

Autism Spectrum Disorder (ASD) is a complex neurodevelopmental condition characterized by deficits in social communication, repetitive behaviors, and restricted interests. Despite increasing prevalence, effective therapeutic interventions remain limited. Repetitive transcranial magnetic stimulation (rTMS) has emerged as a promising non-invasive neuromodulation technique; however, its neural mechanisms and clinical efficacy in children with ASD require further investigation.

**Methods:**

This study enrolled 14 children diagnosed with ASD to undergo a structured rTMS intervention. Neuroimaging data—including voxel-based morphometry (VBM) and resting-state functional connectivity (FC)—as well as behavioral assessments were collected before and after the intervention to evaluate changes in brain structure, function, and symptomatology.

**Results:**

Post-intervention analyses revealed significant increases in gray matter volume (GMV) in the cerebellar Vermis, Caudate nucleus, and Postcentral gyrus. Additionally, enhanced functional connectivity was observed between the Fusiform gyrus, Temporal cortex, Frontal cortex, and Precuneus. Correlation analyses indicated that these neuroimaging changes were significantly associated with improvements in behavioral scores.

**Discussion:**

These findings suggest that rTMS may exert therapeutic effects in children with ASD by modulating cerebellar development and cognitive control networks. The observed structural and functional brain changes support the potential utility of rTMS as a neuromodulatory intervention for ASD. Further studies with larger cohorts are needed to confirm these preliminary results and elucidate the mechanisms underlying rTMS-induced symptom improvement.

## 1 Introduction

Autism Spectrum Disorder (ASD) encompasses a range of complex neurodevelopmental conditions characterized by persistent difficulties in communication, social interaction, and behavior (Zhuang et al., 2023; Ostrowski et al., 2024). The ASD spectrum includes several subtypes, such as Childhood Autism (often simply referred to as autism), Asperger’s Syndrome, and unspecified pervasive developmental disorders, with autism being the most commonly diagnosed (Wang et al., 2024). The core symptoms of ASD often manifest early in life and include speech and communication difficulties, impaired social interaction, repetitive behaviors, and restricted or highly specific interests. These symptoms can vary greatly among individuals, contributing to the diverse presentation of ASD.

The etiology of ASD remains an area of active research and debate. While genetic factors are consistently implicated, environmental influences also contribute significantly, suggesting a multifactorial origin for the disorder (Chen et al., 2023). Studies have indicated that complex interactions between genes and environmental exposures during prenatal and early postnatal development may contribute to neurobiological abnormalities associated with ASD. Recent research using the Liang information flow method has revealed altered hierarchical brain connectivity patterns in ASD, with significant disruptions in stepwise causal connections across multiple brain networks (Sun et al., 2025). These abnormalities include altered connectivity patterns, disrupted neural circuitry, and imbalances in cortical excitation and inhibition. Emerging evidence suggests that the neural changes associated with ASD may begin in the fetal stage, as seen in studies examining differences in cortical thickness and white matter integrity among infants with a higher genetic risk for ASD (Pietropaolo and Provenzano, 2022). These findings underscore the importance of early detection and intervention, as such efforts may alter developmental trajectories and reduce symptom severity (Chen et al., 2020). Despite advances in understanding ASD, its precise mechanisms remain elusive, presenting ongoing challenges in both diagnosis and treatment. Current therapeutic interventions often involve behavioral therapy, speech and language therapy, and educational support, yet the prognosis for individuals with ASD varies and can be limited due to the persistent nature of the core symptoms.

In recent years, researchers have explored novel therapeutic approaches for ASD, particularly focusing on neurobiological interventions that aim to modify underlying neural mechanisms. Transcranial magnetic stimulation (TMS) is one such non-invasive intervention that has garnered growing interest due to its potential to modulate brain activity (Desarkar et al., 2024; Tingting et al., 2025). TMS works by delivering brief, magnetic pulses to targeted brain regions, inducing changes in the membrane potential of cortical neurons (Pedersen and Zalesky, 2021). This modulation not only impacts neuronal activity but also affects broader brain metabolism and function, thereby eliciting physiological and biochemical responses. Advances in TMS technology over the past few decades have significantly expanded its applications in neuroscience, including its use as a diagnostic tool and therapeutic intervention. Single-pulse TMS is typically used to explore brain function, while repetitive transcranial magnetic stimulation (rTMS) is employed to achieve more sustained modulation of brain activity (Klomjai et al., 2015; Jannati et al., 2023). Recent findings from preclinical models have shown that rTMS can reverse ASD-like behaviors, supporting its translational therapeutic relevance (Afshari et al., 2024).

Initially developed as a tool for investigating neural circuitry, rTMS has evolved into a well-established treatment modality for several psychiatric disorders. Since the late 1980s, research has demonstrated that TMS, when combined with electrostimulation therapy, can elicit heightened emotions in healthy individuals, paving the way for its application in psychiatric treatment (Bickford et al., 1987). Consequently, an expanding body of research has explored the application of TMS in treating diverse psychiatric disorders (Stultz et al., 2020; Mizutani-Tiebel et al., 2022), leading to FDA approval for its use in depression treatment in the United States (Koutsomitros et al., 2021). The non-invasive nature of rTMS, along with its ability to specifically target neural circuits implicated in psychiatric conditions, makes it a promising therapeutic approach for ASD as well.

Research on rTMS in ASD has gained momentum, as emerging evidence suggests that this intervention may positively influence repetitive behaviors, social functioning, and executive functioning in individuals with ASD (Barahona-Correa et al., 2018). Some studies have explored variations in stimulation parameters, such as frequency, duration, and target areas of the brain, to optimize rTMS outcomes in ASD treatment. Low-frequency rTMS (typically 1 Hz) has emerged as a promising non-invasive neuromodulation technique for addressing core symptoms of ASD (Sebastianelli et al., 2017). For instance, low-frequency rTMS has been shown to reduce repetitive behaviors and improve social responsiveness in adults with ASD (Enticott et al., 2014), and stimulation targeting the motor cortex has also demonstrated efficacy in alleviating repetitive behaviors (Oberman et al., 2015). These findings collectively support the potential therapeutic utility of low-frequency rTMS in ASD and provide the neurobiological rationale for the present study’s stimulation parameters. Neuroimaging studies have offered further insights into the potential mechanisms underlying the effects of rTMS in ASD. For instance, Masuda et al. (2019) identified cortical excitation/inhibition imbalances as a critical factor contributing to the diverse clinical symptoms of ASD. Recent studies also suggest that rTMS may modulate both neural activity and gut-brain interactions, contributing to multisystem improvements in ASD (Feng et al., 2024). Furthermore, Riva et al. (2013) found that children with low-functioning ASD exhibit reduced cerebellar gray matter volume (GMV) compared to typically developing children, with these structural differences correlated with the severity of cognitive and emotional impairments.

Despite the promising findings regarding rTMS interventions and their effects on brain structure and function, there remains a significant gap in understanding how these neural changes correspond to behavioral improvements. To address this gap, the present study aimed to investigate the correlation between changes in brain structure and function and behavioral improvements following rTMS intervention in children with ASD. This study recruited 14 children diagnosed with ASD to receive rTMS treatment in an open-label, single-arm design. Behavioral assessment scores and neuroimaging data were collected both before and after the intervention to evaluate changes in brain activity and structure. Based on previous evidence of altered cortical excitability and atypical brain connectivity in individuals with ASD, we hypothesized that low-frequency rTMS applied to the left dorsolateral prefrontal cortex (DLPFC) would induce measurable changes in brain structure and functional connectivity and that these neuroimaging alterations would be associated with improvements in behavioral outcomes.

## 2 Materials and methods

### 2.1 Participants

A total of 14 children (aged: 3–8 years) diagnosed with ASD were recruited for this study. All participants were enrolled in the Children’s Rehabilitation Department and outpatient clinics of Sichuan Rehabilitation Hospital (Sichuan Bayi Rehabilitation Center) in Sichuan Province, China. Exclusion criteria included the presence of other mental or neurological disorders, a history of significant trauma or physical illnesses, contraindications to magnetic resonance imaging (MRI), or evidence of structural brain abnormalities on MRI. Children with prior exposure to non-invasive brain stimulation therapies were also excluded. The study was registered in the Chinese Clinical Trials Registry (ChiCTR2200061683) and was approved by the research ethical committee of the University of Electronic Science and Technology of China. The purpose, protocols, and potential risks and benefits of the study were thoroughly explained to the parents or legal guardians, and written informed consent was obtained from all participants’ guardians.

Due to missing or poor-quality neuroimaging data and incomplete behavioral assessment scores, some subjects were excluded from specific analyses. Finally, 11 participants were included in the behavioral and structural MRI (sMRI) analysis, and 7 participants were retained for the functional MRI (fMRI) analysis.

### 2.2 rTMS protocol

Before to rTMS application, participants underwent a preliminary evaluation to assess baseline motor function and ensure safety eligibility. This evaluation included a neurological examination to rule out contraindications, such as a history of seizures, metallic cranial implants, or other conditions that could interfere with magnetic stimulation (Rossi et al., 2021). The target site for stimulation was the DLPFC, identified at the F3 electrode position using the 10–20 EEG system. This site was selected based on prior studies (Lefaucheur et al., 2020), and was stimulated using the Yiruide YRDCCY-1 device equipped with a figure-eight--shaped double-coil. To enhance participant comfort and reduce fatigue, short breaks were provided between stimulation trains. Participants were closely monitored for adverse effects, such as headaches, scalp discomfort, or mood changes, which are commonly reported with rTMS (McCarthy et al., 2023). Safety protocols included the use of ear protection to reduce exposure to device noise and regular monitoring of vital signs during each session. If participants reported discomfort or adverse reactions, stimulation parameters such as intensity or frequency were adjusted accordingly, ensuring adherence to ethical standards and participant safety (Rossi et al., 2021).

The Resting Motor Threshold (RMT) was determined individually for each participant to calibrate the appropriate stimulation intensity. The RMT was defined as the minimum single-pulse magnetic field intensity required to elicit a visible twitch in the first dorsal interosseous muscle. The rTMS intensity was set at 100% of the participant’s RMT, using a low-frequency protocol of 1 Hz (one pulse per second). Each session included a total of 160 pulses, delivered in 8 trains of 20 pulses each, with an inter-train interval of 20–30 s. Participants received rTMS five times per week for four consecutive weeks, for a total of 20 sessions.

In addition, stimulation parameters were individualized based on each participant’s RMT to account for variability in cortical excitability, thereby enhancing the precision and effectiveness of the intervention (Daskalakis et al., 2008). The rTMS, particularly low-frequency (1 Hz) stimulation, has been shown to produce long-term depression (LTD)-like effects, leading to reduced cortical excitability (Lefaucheur et al., 2020). This mechanism is relevant to ASD, where cortical hyperexcitability is thought to underlie core features such as repetitive behaviors and sensory over-responsivity (Casanova et al., 2002; Rubenstein and Merzenich, 2003). The DLPFC a region involved in executive control and inhibitory function, has consistently shown structural and functional abnormalities in individuals with ASD (Schmitz et al., 2006; Uddin et al., 2013). Therefore, targeting the left DLPFC with 1 Hz rTMS at 100% RMT may help restore inhibitory balance in prefrontal circuits, contributing to behavioral improvements (Sokhadze et al., 2010; Oberman et al., 2015). This low-frequency stimulation approach is consistent with prior research indicating that low-frequency rTMS reduces cortical excitability, potentially offering therapeutic benefits for conditions involving cortical hyperexcitability (Lefaucheur et al., 2020).

The overall protocol was designed to maximize participant adherence by incorporating scheduling flexibility and minimizing participant burden, thus reducing dropout risk. Behavioral and neuroimaging assessments were conducted before, midway through, and after the intervention to monitor changes in behavior and cortical activity, enabling a comprehensive evaluation of treatment effects over time. Follow-up assessments were also conducted post-intervention to examine the persistence of rTMS-induced effects and detect any delayed adverse events. These procedures ensured strict compliance with safety standards while optimizing the therapeutic potential of rTMS for modulating neural function in ASD.

### 2.3 Clinical assessment

During the 3 days before treatment initiation and the 3 days following its completion, participants underwent comprehensive assessments using standardized tools to evaluate changes in both core and associated symptoms of ASD. The primary instruments included the Autism Behavior Checklist (ABC), the Social Responsive Scale-Second Edition (SRS-2), and the Repetitive Behavior Scale-Revised (RBS-R), all of which were administered by trained clinicians under controlled conditions (See Table 1 for specific score statistics). This measure was selected based on their demonstrated reliability, validity, and sensitivity in detecting symptom changes in individuals with ASD.

**Table 1:** Demographic and clinical behavioral scale scores of pre- and post-rTMS intervention in ASD.

	Pre-rTMS (*n* = 11)	Post-rTMS (*n* = 11)	*t* value	*p* value
Sex(M/F)	10/1	10/1	–	–
Age	4.2 ± 1.3	4.2 ± 1.3	–	–
SRS	104.4 ± 33.1	95.4 ± 26.4	0.79	0.446
ABC	63.0 ± 39.3	51.3 ± 27.5	1.26	0.238
RBS	21.6 ± 17.6	13.3 ± 8.9	1.77	0.111

ABC, Autism Behavior Checklist. SRS, Social Responsive Scale. RBS, Repetitive Behavior Scale-Revised. M, Male. F, Female.

Caregivers also participated in structured interviews conducted before and after the intervention, providing qualitative insights into changes in behavior, social interactions, and daily routines (Lord et al., 2022). These interviews contributed to a more contextualized interpretation of observed behavioral changes, taking into account external factors such as school transitions, family dynamics, and environmental influences.

By implementing this multi-dimensional assessment strategy, this study aimed to deliver a comprehensive evaluation of both the core symptoms and broader functional outcomes of rTMS treatment. This rigorous design supports a more nuanced understanding of how neurobiological interventions may affect various dimensions of ASD, offering valuable insights into both the efficacy and potential limitations of the therapeutic approach.

### 2.4 sMRI and fMRI image acquisition and preprocessing

In this study, the sMRI and fMRI scans were conducted within 3 days before the initiation of rTMS treatment and within 3 days after the completion of 20 rTMS sessions. Thirty minutes before the scanning, a licensed physician orally administered 10% chloral hydrate (0.5 mL/kg) to induce sedation in children with autism. The children commenced the scan after entering a state of sleep. The sedative effect and safety of oral chloral hydrate in children before MRI examinations have been widely reported (Ronchera-Oms et al., 1994).

All MRI scans were performed using the GE Discovery MR750 3.0 T superconducting magnetic resonance scanner at the Neuroinformatics Key Laboratory of the Ministry of Education, University of Electronic Science and Technology of China. High-resolution images were acquired using a Gradient-echo Echo-planar Imaging (GE-EPI) sequence. For the sMRI, scanning parameters were: repetition time (TR) = 6.008 ms, echo time (TE) = 1.964 ms, flip angle = 12°, inversion time = 450 ms, matrix size = 256 × 256, slice thickness = 1 mm, gap = 1 mm, total slices = 156. For the resting-state fMRI, parameters were: TR = 2000 ms, TE = 30 ms, flip angle = 90°, in-plane matrix size = 64 × 64, slice thickness = 3 mm, gap = 4 mm, total slices = 205.

sMRI data were preprocessed using the SPM12 software package.^1^ The preprocessing workflow included the following steps: (1) spatial normalization, aligning all MRI data into a standard stereotactic space to correct for individual anatomical differences; (2) segmentation of brain tissues into gray matter, white matter, and cerebrospinal fluid based on voxel intensities values (Ji et al., 2019); (3) Spatial smoothing of segmented images to improve the sensitivity of subsequent statistical analysis; and (4) Voxel-wise comparison of regions of interest (ROIs), as gray matter, between groups (Zheng et al., 2020).

fMRI data were preprocessed using the DPABI software package,^2^ which is built upon SPM12 and specifically optimized for fMRI data analysis (Cong et al., 2023). The preprocessing steps were as follows to ensure the accuracy and reliability of subsequent functional connectivity (FC) analyses: (1) Removal of the first 10 volumes to allow for signal stabilization; (2) Slice timing correction; (3) Realignment for head motion correction; (4) Spatial normalization to standard anatomical space; (5) Spatial smoothing using an 8 mm full-width at half maximum (FWHM) a Gaussian kernel; (6) Detrending and filtering. The time series were bandpass filtered (0.01–0.1 Hz) to isolate frequencies relevant to spontaneous neuronal activity and FC. To reduce motion-related artifacts, functional images were realigned and scrubbed based on framewise displacement (FD), with volumes exceeding 0.5 mm FD excluded from analysis. Participants with mean FD > 0.3 mm or with more than 20% of timepoints flagged were excluded. Spatial smoothing was performed using an 8 mm FWHM Gaussian kernel to enhance signal-to-noise ratio, although we acknowledge that smaller kernels may better preserve regional specificity in pediatric populations (Al-Fatly et al., 2023; Candemir, 2023).

### 2.5 Voxel-based morphometry analysis

We employed the CAT12 toolbox for VBM analysis to identify regional differences in brain tissue components while minimizing the influence of macroscopic anatomical variability and spatial orientation. Following the normalization of all structural images to a common stereotactic space, the images were segmented into GM, WM, and CSF, with voxel values modulated to reflect the volume of the brain voxel. Smoothed GM images were then subjected to statistical analysis to detect significant differences between experimental groups. Post-processing quality checks were conducted to address potential artifacts and to ensure accurate segmentation and normalization. For a more detailed examination of GM, mean group GM masks were generated by averaging the normalized GM masks across all subjects. Regions showing abnormal GMV in the VBM analysis were subsequently used as Regions of Interest (ROIs) to assess FC with voxels across the whole brain.

### 2.6 Whole-brain FC analysis

Whole-brain FC analysis was conducted using seed regions identified from prior VBM results, which showed significant differences in GMV. These regions served as seeds for computing FC with the rest of the brain (Yin et al., 2025). The FC calculation procedure included the following steps: (1) Defining Seed Regions: ROIs were defined based on brain areas exhibiting significant GMV differences in the VBM analysis. Each ROI was modeled as a sphere with a 6 mm radius, centered on the peak coordinate of the corresponding GMV cluster; (2) Extracting of Seed Time Series: The time series from each seed region was extracted for subsequent correlation analysis; (3) Voxel-Wise Correlation Mapping: Pearson correlation coefficient were calculated between the seed time series and the time series of every voxel across the whole brain to generate voxel-wise FC maps; (4) Fisher Z Transformation: The resulting correlation coefficients were transformed into Z-scores using Fisher’s r-to-z transformed to improve distributional normality; (5) Group-Level Analysis: Group level statistical analyses were conducted to compare FC patterns across conditions. Each participant’s Z-score map was entered into the group analysis to identify significant connectivity differences associated with the predefined ROIs.

### 2.7 Statistical analysis

A *post-hoc* power analysis was conducted using G*Power (v3.1) to assess the sensitivity of the current study. With a sample size of 14 participants, an alpha level of 0.05, and a two-tailed paired-samples t-test design, the analysis showed that the study achieved 80% power to detect a large effect size (Cohen’s d = 0.81). This suggests the study was sufficiently powered to identify robust intervention effects, though it may have been underpowered for detecting more subtle changes.

Statistical analyses were conducted using MatLab software. Behavioral scale scores were compared using paired sample t-tests to assess between-group differences before and after the rTMS intervention. Additionally, paired sample t-tests were used to evaluate within-group changes in behavioral scores across time points.

For neuroimaging data, voxel-wise paired sample t-tests were performed to examine pre- and post-rTMS differences in GMV among children with ASD. To control for multiple comparisons, Gaussian Random Field (GRF) correction was applied, with thresholds set at a voxel *p*-value < 0.005 and cluster-level *p*-value < 0.005. Following the identification of regions showing significant GMV changes, mean signal intensities were extracted from these regions, and Pearson’s correlation analyses were conducted to examine associations between GMV alterations and improvements in behavioral outcomes. This approach aimed to elucidate the structural basis underlying behavioral improvements following rTMS in children with ASD.

For the whole-brain FC analysis, voxel-wise paired samples t-tests were similarly conducted to assess changes in FC pre- and post-rTMS within each group, focusing on specific brain clusters as well as global connectivity patterns. A Region of Interest (ROI) based approach was used to identify significant FC differences, and average changes in FC values across these regions were calculated. Subsequent correlation analyses were performed to explore associations between FC alterations and changes in behavioral scale scores, providing insight into the potential contribution of rTMS-induced connectivity changes to behavioral improvements in ASD.

### 2.8 Correlation analysis

Although group-level comparisons revealed no statistically significant differences in ABC, SRS, or RBS scores before and after rTMS treatment (all *p*-values > 0.05), some participants showed observable reductions in symptom severity. Given the known individual variability in rTMS response among individuals with ASD, we conducted exploratory correlation analyses to examine whether changes in neuroimaging measures (GMV and FC) were associated with individual-level behavioral changes. This approach may offer insight into the neural mechanisms that underlie behavioral variability, even in the absence of significant group-level treatment effects (Enticott et al., 2012; Oberman et al., 2015). Pearson correlation analysis was performed to explore associations between changes in structural and functional neuroimaging metrics (i.e., GMV and FC) and changes in behavioral scores (i.e., ABC and RBS) in children with ASD. The ABC scale was used to assess overall behavioral symptoms, while the RBS scale evaluated stereotyped behaviors and restricted interests. Correlation were considered statistically significant at *p* < 0.05.

## 3 Results

### 3.1 Differences in GMV between pre- and post-rTMS scans

**Figure 1:**
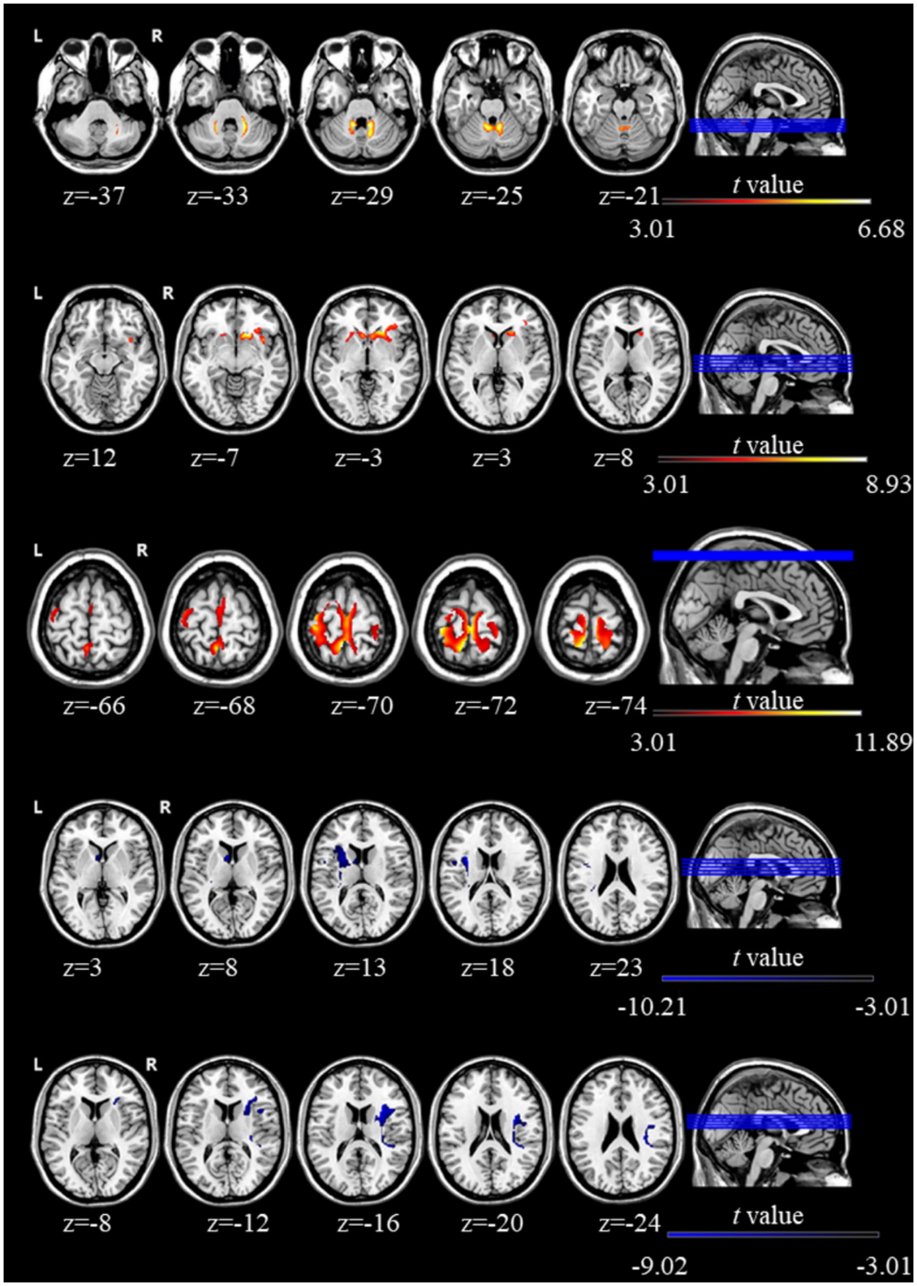
Regions showing significant changes in GMV between the pre- and post-rTMS intervention in ASD.

Compared to pre-rTMS condition, significant increases in GMV were observed in the cerebellar vermis lobules IV-V, right caudate, and left postcentral gyrus, while significant decreases were found in the left caudate and right insula following rTMS stimulation (GRF, *p* < 0.005). Figure 1 illustrates these GMV differences between pre- and post-rTMS scans in children with ASD. A cluster-level threshold of *p* < 0.005 and a voxel size threshold greater than 50 voxels were applied. Specific regions showing altered GMV are detailed in Table 2.

**Table 2:** Changes in brain GMV in ASD before and after rTMS intervention.

Brain region	Cluster size	*t* value	MNI coordinates		
			*x*	*y*	*z*
Cerebellar vermis lobules IV-V	1,064	6.68	14	−45	−30
Right caudate	1,424	8.93	18	21	−6
Left postcentral gyrus	2,227	11.89	−3	−50	69
Left caudate	1,202	−10.21	−30	3	18
Right insula	1,453	−9.02	30	5	18

MNI refers to the Montreal Neurological Institute; X, Y, Z represent the coordinates of peak locations in MNI space; all brain regions were corrected for GRF (*p* < 0.005).

### 3.2 Differences in whole-brain FC between pre- and post-rTMS scans

**Figure 2:**
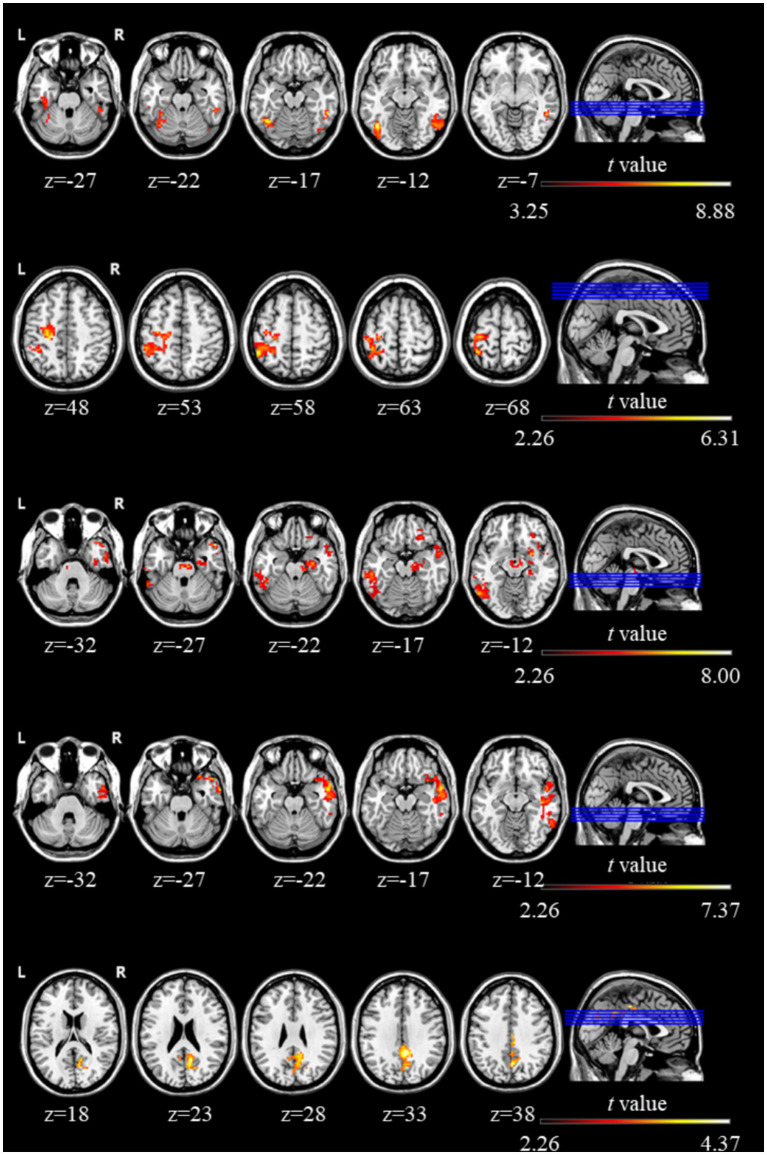
Regions showing significant changes in whole-brain FC between the pre- and post-rTMS intervention in ASD.

Relative to pre-rTMS condition, significant increases in whole-brain FC were observed in the left fusiform gyrus, left inferior temporal gyrus, left postcentral gyrus, right inferior frontal gyrus, right middle temporal gyrus, and right precuneus following rTMS stimulation (GRF, *p* < 0.005). Figure 2 presents the brain regions showing significant FC changes between pre- and post-rTMS conditions in children with ASD. A cluster-level significance threshold of p < 0.005 and a voxel size threshold >50 voxels were applied. Full details of the altered FC regions are listed in Table 3.

**Table 3:** Changes in whole-brain FC of ROIs before and after rTMS intervention.

Cluster	Brain region	Cluster size	*t* value	MNI coordinates		
				*x*	*y*	*z*
Cluster 1	Left fusiform gyrus	177	8.88	−42	−60	−15
	Right inferior temporal gyrus	149				
Cluster 2	Left postcentral gyrus	425	6.31	−21	−24	48
Cluster 3	Right temporal lobe	620	8.00	51	18	−27
	Right inferior frontal gyrus	558				
	Left inferior temporal gyrus	427				
Cluster 4	Right middle temporal gyri	951	7.37	45	0	−42
Cluster 5	Right precuneus	370	4.38	3	−24	45

### 3.3 Correlation analysis

**Figure 3:**
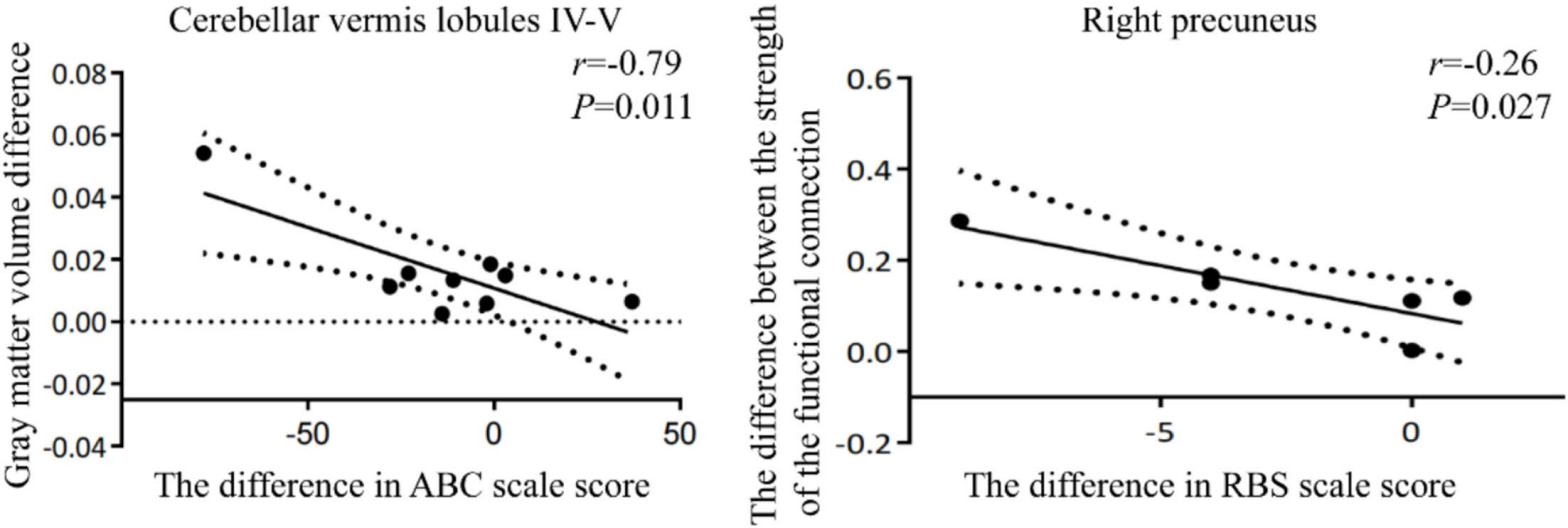
The correlations between changes in GMV and FC coupling changes and improved clinical symptoms in ASD. ABC, Autism Behavior Checklist. RBS, Repetitive Behavior Scale-Revised.

Correlations between changes in GMV and FC and corresponding changes in behavioral scores were examined in children with ASD. The change in GMV of cerebellar vermis lobules IV-V region was negatively correlated with the change in ABC scores (*r* = −0.79, *p* = 0.011), while the change in FC of the right precuneus was significantly negatively correlated with the change in RBS scores (*r* = −0.26, *p* = 0.027). The corresponding scatter plots are presented in Figure 3. Descriptive statistics of behavioral scores before and after the intervention are presented in Table 1.

## 4 Discussion

This study aimed to investigate the neural mechanisms underlying the improvement of ASD symptoms following rTMS intervention. The main findings are summarized as follows: (1) sMRI: rTMS led to increased GMV in regions including the cerebellar vermis lobules IV-V, right caudate, and left postcentral gyrus, and decreased GMV in the left caudate and right insula; (2) FC analysis: Children with ASD showed increased FC in several regions post-rTMS, including the left fusiform gyrus, left postcentral gyrus, right temporal lobe, left inferior and right middle temporal gyri, right inferior frontal gyrus, and right precuneus; (3) Correlation Analysis: Increase in vermis GMV were negatively correlated with ABC scores, suggesting a positive effect of rTMS on cerebellar development in children with ASD; (4) Reduction in Repetitive Behaviors: Enhanced FC in the prefrontal cortex and across the whole-brain was significantly negatively correlated with RBS scores, indicating a reduction in repetitive behaviors. This study utilized VBM and FC analysis to investigate structural and functional changes in children with ASD, consistent with previous research methodologies (Anteraper et al., 2020). These analyses target different brain characteristics, but their interplay highlights the complexity of ASD. rTMS intervention demonstrated notable improvements in behavioral abnormalities among ASD children, and these improvements were significantly correlated with alterations in both brain structure and function. These findings provide evidence supporting the neuromodulatory effects of rTMS in ASD and contribute to the growing understanding of the relationship between brain structure, FC, and behavioral outcomes in this population.

Our selection of 1 Hz rTMS over the left DLPFC was informed by its capacity to modulate cortical excitability via LTD-like mechanisms. Given the well-documented cortical hyperexcitability in ASD (Casanova et al., 2002), this inhibitory stimulation paradigm may help normalize dysregulated prefrontal function. The DLPFC is implicated in cognitive control and behavioral inhibition - functions often disrupted in ASD - making it a plausible target for reducing repetitive and inflexible behaviors. The observed correlations between FC changes and behavioral improvements further support the mechanistic relevance of this protocol.

Regarding structural imaging findings, the cerebellar vermis lobules IV-V region, part of the cerebellum, is known to play a key role in motor coordination and cognitive processing (Saito, 2023). Consistent with Webb et al., which reported reduced vermis volume in children with ASD compared to typically developing peers (Webb et al., 2009), the present study observed an increase in the vermis GMV following rTMS intervention. This increase was negatively correlated with ABC scores, suggesting that rTMS may promote cerebellar development in children with ASD. While the cerebellar vermis has been implicated in motor and cognitive processes, this finding should be interpreted as an association rather than direct evidence of functional improvement following rTMS. The left postcentral gyrus, part of the somatosensory cortex involved in tactile information processing (Kropf et al., 2019), has been consistently reported to exhibit reduced GMV in children with ASD (Mizuno et al., 2019). The current finding of increased GMV in this area post-rTMS may reflect a reversal of such structural deficits. In addition, the observed increase in right caudate volume and decrease in left caudate volume are in line with earlier studies showing age-related caudate enlargement in ASD (Lange et al., 2015; Zuo et al., 2019), suggesting a modulatory effect of rTMS on caudate development. The Insula, implicated in social–emotional processing and interoception (Nomi et al., 2019), also exhibited volume changes following rTMS. These structural alterations may relate to improved emotional and social functioning. Collectively, our findings support previous reports (Barahona-Correa et al., 2018) highlighting the positive effects of rTMS on repetitive behaviors and executive function in ASD. The observed GMV changes in the Vermis, left postcentral gyrus, Caudate, and right insula suggest potential structural mechanisms through which rTMS may alleviate core ASD symptoms.

In terms of functional imaging, this study revealed increased whole-brain FC in children with ASD following rTMS intervention, particularly involving the left fusiform gyrus, left postcentral gyrus, right temporal lobe, inferior temporal gyrus, right middle temporal gyrus, right inferior frontal gyrus, and right precuneus. Prior resting-state fMRI studies have consistently demonstrated reduced FC among regions of the social brain in individuals with ASD (Sato and Uono, 2019). Additionally, Feng et al. reported that temporal variability in FC involving the Temporal lobe and Precuneus was associated with social behavioral performance in ASD (Feng et al., 2023). Since FC reflects the synchronization of neuronal activity between brain regions, changes in these networks may underlie improvements in social and sensory functioning. Notably, the Fusiform, right temporal lobe, and right inferior frontal gyrus regions are key areas implicated in social cognition and face processing (Kuno-Fujita et al., 2020). Dysfunction in these areas may contribute to the characteristic social impairments observed in ASD. Meanwhile, the left postcentral gyrus is primarily responsible for somatosensory information, and both the inferior temporal gyrus and the right precuneus have been associated with sensory processing. Therefore, the FC enhancements observed in these regions may reflect a normalization of sensory and social networks following rTMS treatment.

The left fusiform gyrus is a crucial region for face processing and social cue processing (Kuno-Fujita et al., 2020). Individuals with ASD frequently exhibit impairments in recognizing faces and interpreting social stimuli, and numerous studies have reported hypoactivity in the left fusiform gyrus in this population, underscoring its role in social cognition (Goodwill et al., 2023). Dysfunction in this region may contribute to deficits in visual attention and social interaction commonly observed in ASD. The temporal lobe, comprising several subregions such as the inferior, middle, and superior temporal gyri, is involved in processing visual and auditory stimuli, memory encoding, and emotional regulation (Catani, 2022). The inferior temporal gyrus plays a central role in visual and face recognition, while the middle temporal gyrus is associated with auditory processing and language comprehension (Zachlod et al., 2022). Qin et al. reported significantly reduced FC in the temporal lobe of children with ASD (Qin et al., 2022), and early behavioral studies have documented impairments in speech recognition, particularly for unfamiliar auditory stimuli (Boucher et al., 1998). However, recognition of familiar sounds in ASD tends to be preserved (Schelinski et al., 2014). In this study, rTMS enhanced FC in multiple temporal lobe regions, including the superior, middle, and inferior temporal gyri, suggesting that stimulation may help restore auditory network function and improve speech perception in preschool-aged children with ASD. Recent TMS-EEG work also supports this, showing rTMS-induced modulation of brain connectivity in individuals with ASD (Yang et al., 2024). These improvements may, in turn, contribute to enhanced social communication abilities, although no direct measures of speech perception were collected in the present study.

The left postcentral gyrus, which comprises the primary somatosensory cortex, is responsible for processing tactile sensory input (Kropf et al., 2019). Children with ASD often display sensory processing differences, including hypersensitivity to tactile stimuli (Wang et al., 2023), and left postcentral gyrus has also been implicated in motor coordination (Zimmerman et al., 2014). Previous studies have reported weakened FC between the cerebellum and left postcentral gyrus in individuals with ASD, which may contribute to impairments in motor function and coordination (Ramos et al., 2018). While this region is primarily known for its sensory and motor functions, it has also been associated with social and emotional processing. For instance, Fittipaldi et al. found that experiences of envy in ASD were linked to increased activation in the left postcentral gyrus, suggesting a possible role in mentalization and emotional experience (Fittipaldi et al., 2023). The right inferior frontal gyrus, specifically the right inferior frontal gyrus is closely associated with motor inhibition control (Schaum et al., 2021). Lesions in this region may delay the initiation of inhibitory responses, though execution may remain intact (Choo et al., 2022). Given that motor difficulties affect approximately 80% of individuals with ASD, the inferior frontal gyrus has been implicated in both motor and social-cognitive deficits. Studies have shown reduced connectivity between this region and brain areas involved in mentalizing processes (Jayashankar et al., 2023). The right precuneus is a key hub for visual processing and complex cognitive operations such as self-referential thought and spatial cognition (Dadario and Sughrue, 2023). In children with ASD, atypical connectivity involving the precuneus and visual/prefrontal cortices has been associated with difficulties in communication and language, and such patterns are typically absent in neurotypical children (Xiao et al., 2023).

Correlation analysis demonstrated that increased GMV in the cerebellar Vermis was significantly negatively associated with ABC scores in children with ASD. The cerebellum plays a pivotal role in motor coordination, balance, and higher-order cognitive functions (Saito, 2023). Structural alterations in this region may thus influence performance across motor and cognitive domains. These findings suggest that rTMS may contribute to improvements in abnormal cerebellar development, which in turn are linked to reductions in core ASD symptoms. Additionally, increased FC between the right precuneus and the whole brain was significantly negatively correlated with RBS scores. The right precuneus is a central node involved in cognitive control, self-regulation, and introspective processing (Hebscher et al., 2020). Reduced FC in this region has been associated with difficulties in executive functioning and behavioral regulation in ASD, which may underlie the severity of repetitive and restricted behaviors. The present findings imply that rTMS may enhance functional integration in the precuneus, contributing to reductions in stereotyped behaviors.

This study has several limitations that warrant consideration. First, the small sample size may have impacted the study’s statistical power, reducing its ability to detect subtle effects of rTMS on brain function and structure. Given the challenges of conducting rTMS and MRI procedures in young children with ASD, small samples are common in the current literature. Future research should focus on gradually accumulating larger, multicenter datasets to validate and extend the present findings. Second, this study does not provide sufficient detail on strategies used to control for multiple comparisons, which may compromise the reliability of the results. The absence of quantitative evaluations of intervention effects, coupled with the lack of long-term follow-up data, further limits the interpretation of the study’s findings. In addition, the absence of a sham rTMS control group restricts our ability to isolate the specific neuromodulatory effects of the intervention. Without a control condition, we cannot rule out the possibility that non-specific factors such as placebo effects or participant-clinician interaction contributed to the observed improvements. This limitation should be addressed in future randomized, sham-controlled trials. Third, the sample consisted exclusively of Chinese children with ASD, limiting the cultural and ethnic generalizability of our findings. Differences in sociocultural context, language development, and neurobiological profiles may influence the response to rTMS. Replication in more diverse, multinational cohorts is necessary to establish the broader applicability of these results. In conclusion, although the study offers preliminary evidence regarding the effects of rTMS on brain structure and function in children with ASD, several methodological and interpretive remain. Future research should address these limitations by employing more rigorous experimental designs and comprehensive analytical approaches to generate clinically meaningful insights into rTMS interventions for children with ASD.

## 5 Conclusion

In conclusion, this study presents a comprehensive investigation into the effects of rTMS on brain structure and function in children with ASD. Although conducted with a relatively small sample, the study identified preliminary correlations between pre- and post-intervention neuroimaging changes and behavioral outcomes. The findings reveal significant changes in GMV and FC following rTMS treatment, but the absence of a control group and limited behavioral-clinical linkage calls for cautious interpretation. These results provide exploratory evidence of potential therapeutic effects, which require further validation through larger, sham-controlled trials.

## Data Availability

The datasets presented in this article are not readily available because the data are not publicly available due to privacy or ethical restrictions. Requests to access the datasets should be directed to Tao Zhang, zhangtao1698@xhu.edu.cn.

## Footnotes

^1^ http://www.fil.ion.ucl.ac.uk/spm

^2^ http://rfmri.org/dpabi
